# Recent Advances in Interface Engineering for Electrocatalytic CO_2_ Reduction Reaction

**DOI:** 10.1007/s40820-021-00738-9

**Published:** 2021-10-25

**Authors:** Junjun Li, Sulaiman Umar Abbas, Haiqing Wang, Zhicheng Zhang, Wenping Hu

**Affiliations:** 1 Tianjin Key Laboratory of Molecular Optoelectronic Sciences, Department of Chemistry, School of Science, Tianjin University and Collaborative Innovation Center of Chemical Science and Engineering, Tianjin, 300072 People’s Republic of China; 2 Institute for Advanced Interdisciplinary Research (iAIR), University of Jinan, Jinan, 250022 People’s Republic of China

**Keywords:** Interface engineering, CO_2_ reduction reaction, Electrocatalysis, Heterostructure

## Abstract

Electrocatalytic CO_2_ reduction reaction (CO_2_RR) can store and transform the intermittent renewable energy in the form of chemical energy for industrial production of chemicals and fuels, which can dramatically reduce CO_2_ emission and contribute to carbon-neutral cycle. Efficient electrocatalytic reduction of chemically inert CO_2_ is challenging from thermodynamic and kinetic points of view. Therefore, low-cost, highly efficient, and readily available electrocatalysts have been the focus for promoting the conversion of CO_2_. Very recently, interface engineering has been considered as a highly effective strategy to modulate the electrocatalytic performance through electronic and/or structural modulation, regulations of electron/proton/mass/intermediates, and the control of local reactant concentration, thereby achieving desirable reaction pathway, inhibiting competing hydrogen generation, breaking binding-energy scaling relations of intermediates, and promoting CO_2_ mass transfer. In this review, we aim to provide a comprehensive overview of current developments in interface engineering for CO_2_RR from both a theoretical and experimental standpoint, involving interfaces between metal and metal, metal and metal oxide, metal and nonmetal, metal oxide and metal oxide, organic molecules and inorganic materials, electrode and electrolyte, molecular catalysts and electrode, etc. Finally, the opportunities and challenges of interface engineering for CO_2_RR are proposed.

## Highlights

- This review summarizes current developments in interface engineering for electrocatalytic CO_2_ reduction reaction (CO_2_RR).
- The interface engineering for electrocatalytic CO_2_RR involves the metal–metal interface, metal–metal oxide interface, metal–nonmetal interface, metal oxide–metal oxide interface, organic molecules–inorganic materials interface, electrode–electrolyte interface, and molecular catalysts–electrode interface.
- The opportunities and challenges of interface engineering for CO_2_RR are proposed.

## Graphical abstract

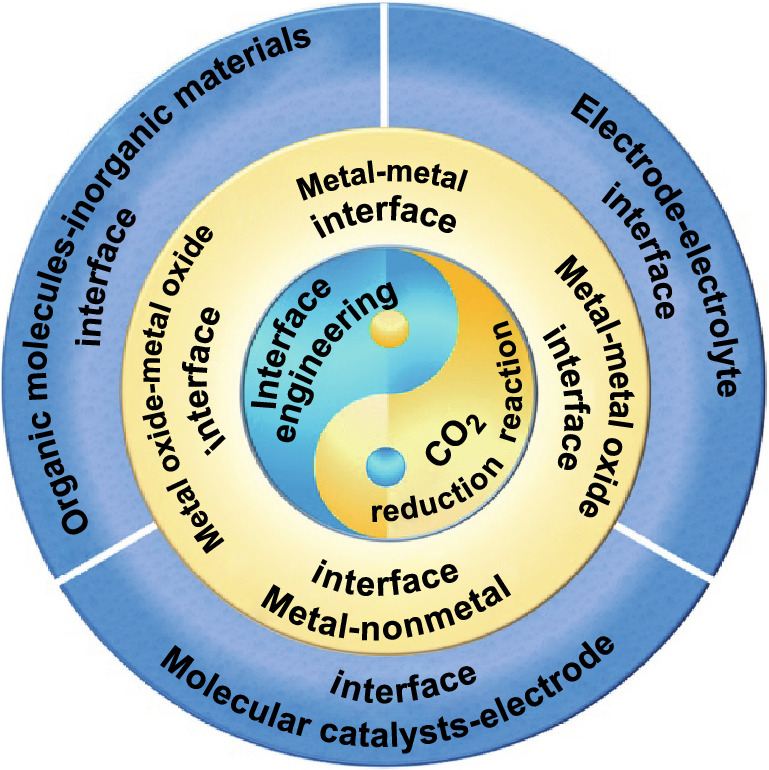

## Introduction

The combustion of fossil fuels into the atmosphere liberates significant volume of greenhouse gases, resulting in the continuous gathering of CO_2_ and an imbalance in the carbon cycle [1–13]. The current overall concentration of CO_2_ in the airspace reached up to 416.96 ppm in 2021 [14]. The overconcentration of CO_2_ in the atmosphere has crucial negative impacts on the climate and environment, such as climate warming, ocean acidification, and glaciers thaw, which will impact the survival and development of human beings seriously [15–18]. Until now, the conversion of CO_2_ to value-added chemicals and fuels is a promising solution to reducing the emission of CO_2_ [19–27]. Among various CO_2_ conversion techniques (electrocatalysis [28–37], photocatalysis [38–45], thermocatalysis [46], biochemical reduction [47, 48], mineralization [49–51], chemical reforming [52–54], etc.), the electrochemical technique has been considered as an effective strategy for its mild operation conditions, clean reaction process, and wide range of reduction products [55–59]. In addition, this process can be powered by renewable energies such as solar, wind, and tide, thus fulfilling the storage of electricity generated from intermittent renewable green energy [60–71]. Therefore, the electrocatalytic CO_2_ reduction (CO_2_RR) offers a sustainable and carbon-neutral route to generate high value-added fuels and feedstocks.

In recent decades, researches about CO_2_RR have been focused on understanding the electrocatalytic pathways, the properties of electrocatalysts, the configuration of electrochemical cell, and the economic feasibility for large-scale production. Specifically, a typical CO_2_RR process mainly includes three steps, i.e., the chemisorption of CO_2_ from electrolyte to the surface of electrocatalyst, the breaking of C–O bonds and/or the formation of C–H bonds through electron transfer and/or protonation process, and the desorption of rearranged product species from the surface of catalyst into electrolyte [72]. However, due to the high bonding energy (750 kJ mol^−1^) of the C=O double bond and the low solubility of CO_2_ in water, CO_2_ reduction is an energy consuming and kinetically slow process [73]. In the step of transfer of electrons and protons, CO/HCOOH, HCHO, CH_3_OH, and CH_4_ are formed correspondingly with the consumption of 2, 4, 6, and 8 electrons during the reaction. In contrast to the C_1_ products, the C_2+_ products are generally formed via a complex carbon–carbon (C–C) coupling reaction. Although exciting advances have been made in the field of CO_2_RR, its development is still suffering from the low yield and unsatisfactory Faradaic efficiency (FE), owing to the chemical inertness of CO_2_ molecule, the sluggish reaction kinetics, the competition between hydrogen evolution reaction (HER) and CO_2_RR, and the scaling relation of the binding energy for reaction intermediates [74, 75]. Energy-efficient, highly selective, and readily available electrocatalysts are highly desired to solve above-mentioned problems, which fundamentally requires the design, modification, optimization of catalyst materials, and the disclosure of significant mechanism through theoretical calculation and in situ spectroscopic analyses.

In recent years, interface engineering has brought novel and exciting possibilities, such as confinement, electronic, and synergistic effects, to improve catalytic properties through intense interactions between different components [76]. An interface is the boundary between two domains that facilitates interactions and synergistic effects among various active actors, resulting in remarkable ability in modulating intermediate adsorption/desorption, managing electron transmission, and mass movement [76, 77]. With the fast advancement of nanotechnology, it is believed that interface engineering would develop into a successful technique to address the important issues and thus to improve catalytic activity, selectivity, and stability.

**Fig. 1:**
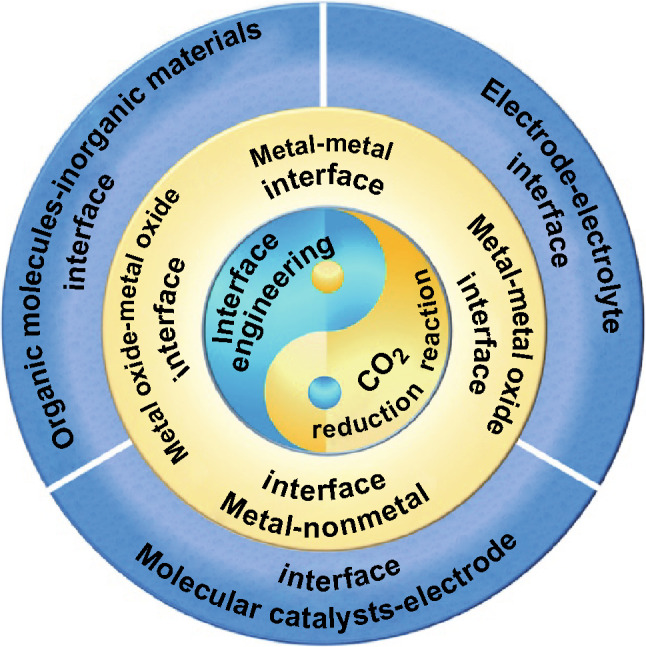
Schematic overview of interface catalysis in electrochemical CO_2_RR covered in this review

The primary purpose of this review is to provide a comprehensive overview of current development in the interface engineering for CO_2_RR from both a theoretical and experimental standpoint, involving interfaces between metal and metal, metal and metal oxide, metal and nonmetal, metal oxide and metal oxide, organic molecules and inorganic materials, electrode and electrolyte, molecular catalysts and electrode, etc. (Fig. 1). Finally, the opportunities and challenges of interface engineering for CO_2_RR are proposed.

## Reaction Pathways and Key Scientific Issues of CO_2_RR

**Scheme 1:**
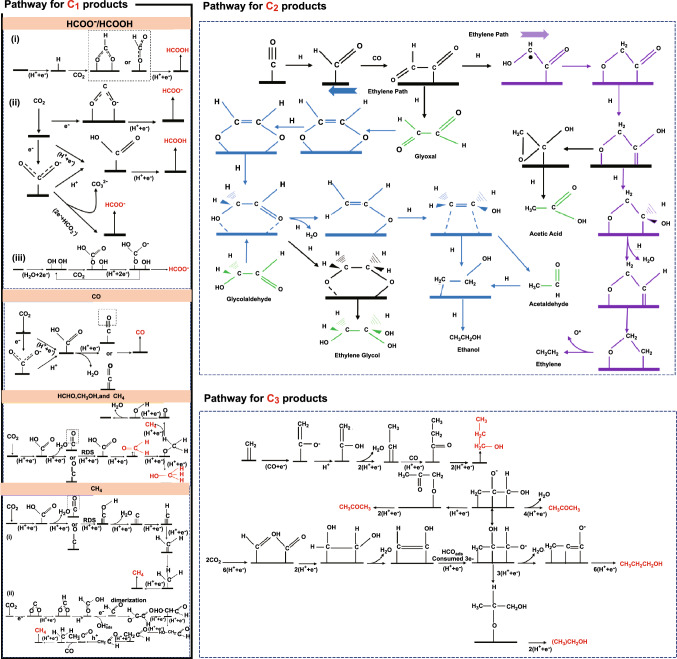
Overview of the possible reaction pathways of CO_2_RR for C_1_, C_2_, and C_3_ products. Reprinted with permission from Ref. [78]

In recent review articles, the measurement system for CO_2_RR including cell configuration, electrochemical measurements and catalytic activity descriptors, products detection, and the techniques for monitoring reaction pathways has been comprehensively proposed. Especially, the reaction mechanism and the pathways for C_1_, C_2_, and C_3_ productions of CO_2_RR have been comprehensively investigated based on in situ characterization and theoretical calculations, although some detailed parts are still controversial. Here, we focus on revealing the key scientific issues encountered in CO_2_RR research by briefly explaining the representative pathways in order to inspire the subsequent studies on CO_2_RR (Scheme 1).

### High Energy Input

Owing to a high bonding energy of about 750 kJ mol^−1^ in C=O bond, CO_2_ molecules have high thermodynamic stability and chemical inertness, indicating that CO_2_RR requires more energy to break the C=O bond. As a result, the starting step of first electron transfer to generate CO_2_^−^ radical usually requires a more negative redox potential of − 1.9 V versus standard hydrogen electrode, which is widely considered as the rate-determining step for CO_2_RR. Besides, CO_2_RR also contains complex reaction processes with multiple electrons and protons transfer, which implies kinetically sluggish processes. For future industrial-scale applications, it is important to develop highly efficient CO_2_RR catalysts to achieve lower energy consumption and faster reaction kinetics.

### Undesirable Competing HER

As mentioned above, CO_2_RR usually requires the participation of protons, and the species in the electrolytes such as water, bicarbonate, hydronium ions, and carbonic acid can be used as proton sources. According to the theoretical calculation results, the binding energy of adsorbed hydrogen needs to match the binding energy of C-binding intermediates for hydrocarbon conversion; otherwise, hydrogen will first combine to form hydrogen gas, resulting in much lower selectivity. As a result, the HER is undesirable and competing over CO_2_RR, and selective HER inhibition is an urgent issue for CO_2_RR.

### Linear Scaling Relation

Based on the available reaction pathways, it is known that the different products generally have the same initial or in-process intermediates. For example, *CO is generally considered to be the common initial intermediate for most of the products including CO, alkanes, and alcohols. And for the pathways of C_2+_ chemicals such as CH_3_COOH, C_2_H_4_, and C_2_H_5_OH, it usually undergoes complex joint *CO–CO or its protonated form from the very initial *CO. Moreover, *CHO is recognized to be the in-process intermediate for CH_4_ and CH_3_OH generation, which is then converted to different final products according to the difference of their binding energy with the catalyst surface, respectively. In addition, there is a linear scaling relation in the binding energies of different intermediates on the catalyst surface. In other words, the binding affinities of different intermediates involved in the reaction are similar, resulting in difficulty in controlling their adsorption and desorption modulation. Therefore, it is very challenging to obtain single products with high FE and high selectivity, especially multi-carbon products.

### Low CO_2_ Concentration Limitations

For CO_2_RR, CO_2_ molecules are used as a carbon source. Normally, CO_2_ gas is continuously passed into the electrolyte, which dissolves and forms a CO_2_-saturated solution. Then, CO_2_ molecules migrate to the cathode surface by convection or diffusion to form CO_2_RR products via proton and electron transfer. CO_2_-saturated KHCO_3_ solution is a commonly used electrolyte, in which the pH in the bulk is in the range of 6.8 to 7.2 and the concentration is estimated to be about 33 mM. Currently, the low CO_2_ solubility and the slow interfacial transport greatly limit the efficiency of CO_2_RR. Increasing the local concentration of CO_2_ on the electrode surface through some strategies such as reaction cell configuration design (e.g., flow cell), electrolyte screening, electrode and electrolyte interface, and electrode and gas interface is important to improve the electrocatalytic efficiency of CO_2_.

## Interface Engineering for CO_2_RR

Among various reported strategies to address the aforementioned problems for CO_2_RR, such as particle size, crystal surface, morphology, and defects, interface engineering has the advantage of being extremely rich and adjustable for regulating reaction processes. From the perspective of a loaded catalyst, the phases that make up the interface can be viewed as the loading phase and the supporting phase. The interface is able to contribute to the dispersion and the stability of both two domains. Moreover, compared to the bulk phase atoms, the atoms located at the interface of the object are in a coordination unsaturated state, which are usually the active sites for the reactions and prone to the physisorption, chemisorption, or direct chemical reactions with other species to generate new species. In addition, the interface shows very significant advantages in regulating bonding energy, electron transfer, transformation and transport of adsorbates and intermediates, which still needs to be studied more carefully. Several principles (the compatibility of materials, electrophilicity of each component, the conductivity after the composition and the variation of electronic distribution on the surface and interface) should be taken into consideration for fulfilling better electrocatalytic performance [79].

### Metal–Metal Interface

Recently, integrating the second metals to construct metal–metal interface has attracted wide attention due to the cooperative effects of bimetal species at the interface [80]. Among all metals, Cu, thanks to its unique electronic properties, has become the most effective monometallic catalyst for CO_2_RR to convert CO_2_ to various hydrocarbons and oxygenates, such as methane, ethylene, methanol, ethanol, and C_3+_ products [81–89]. Moreover, the features of high activity, low toxicity, and high abundance [90] enable the Cu to commercialize CO_2_RR. However, the present issues, such as low selectivity toward a specific product and low FE due to competition with HER and sluggish kinetics, still need to be addressed [76, 81, 84, 85, 90, 91]. In recent years, various interface-related strategies have been devised to enhance its catalytic performance, such as intermetallic compounds [92], heteroatomic doping [68, 93], single Cu atom catalysts [94], core–shell structures [86], and heterostructure [95–97]. Among them, alloying is a general and widespread method to improve catalytic performance [98–100]. However, the intrinsic electronic properties of the component metals will be significantly modified after alloying. By contrast, constructing metal–metal interface can, to some extent, preserve the intrinsic feature of the metals [101], which is in favor of realizing high electrocatalytic performance [101–103].

**Fig. 2:**
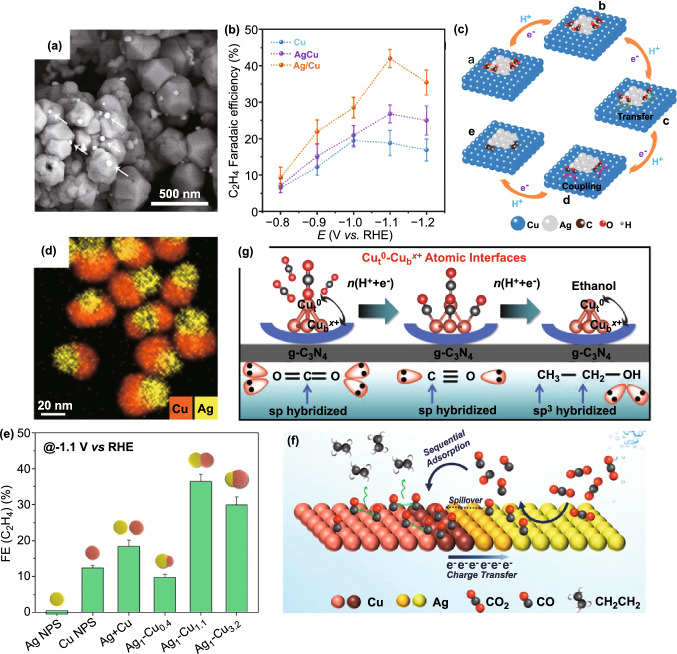
**a** Scanning electron microscope (SEM) image of Ag/Cu_2_O. **b** FE of C_2_H_4_ for Ag/Cu_2_O catalyst. **c** Mechanism for converting CO_2_ to C_2_H_4_ on Ag/Cu interface. Reprinted with permission from Ref. [82]. **d** EDX elemental maps of Ag_1_–Cu_1.1_ NDs. **e** FEs of C_2_H_4_ obtained on different Ag/Cu nanocrystals. **f** The tandem catalysis mechanism of C_2_H_4_ promotion in the Ag–Cu NDs. Reprinted with permission from Ref. [83]. **g** Schematic of mechanism for conversion to CH_3_CH_2_OH on Td-Cu_4_@g-C_3_N_4_. Reprinted with permission from Ref. [108]

For example, Wang et al. constructed exposed Ag/Cu interface by distributing Ag nanoparticles on the surface of Cu nanoparticles (Fig. 2a) [82]. The electrocatalytic performance of four catalysts (Ag/Cu, AgCu alloy, pure Cu, and Ag NPs) was compared for CO_2_RR (Fig. 2b). It is obvious that Ag/Cu catalyst exhibited maximum FE of 42% toward C_2_H_4_ at − 1.1 V versus reversible hydrogen electrode (vs. RHE), obviously superior to the other three catalysts. Further, they proposed a detailed process to explain how the interface facilitates the production of C_2_H_4_. As shown in Fig. 2c, CO, the key intermediate for hydrocarbons, is obtained on Ag atoms after the following two-step electron and proton transfer process:

$${\text{CO}}_{2} + ^{*} + \left( {{\text{H}}^{ + } + {\text{e}}^{ - } } \right) \to {}^{ * }{\text{COOH}}$$

$$^{*} {\text{COOH}} + \left( {{\text{H}}^{ + } + {\text{e}}^{ - } } \right) \to {}^{ * }{\text{CO + H}}_{{\text{2}}} {\text{O}}$$

Then, CO species that transfer to Cu atoms can further couple into C_2_H_4_ via continuous proton–electron transfer. Owing to higher CO binding energy of Cu than that of Ag, CO intermediates accumulate in large quantities at the interface which is in favor of the formation of C–C bond by coupling, i.e., a rate-determining step for C_2+_ production. As for AgCu alloy, the existence of a transition AgCu layer is suggested to depress the process of CO dimerization, consequently resulting in low selectivity of C_2_H_4_.

Almost at the same time, Huang et al. further explored and illustrated interface effect between Cu and Ag for CO_2_RR [83]. They synthesized Ag–Cu nanodimers (NDs) (Fig. 2d) by using Ag nanoparticles as nucleation seeds, which apparently outperformed pure Ag and Cu NPs in terms of FE of C_2_H_4_. Particularly, Ag_1_–Cu_1.1_ NDs (mass ratios) exhibited FE of approximate 40% toward C_2_H_4_ at − 1.1 V versus RHE, a 3.4-fold enhancement compared with pure Cu NPs (Fig. 2e). Combining with previous reports [104–106], they speculated that the Ag domain serves as supplier of CO to adjacent Cu domain, and meanwhile, Cu supplies electron to Ag. Subsequent control experiment that employed Ag + Cu mixture (a physical mixture of Ag NPs and Cu NPs) as catalyst verified their hypothesis with density functional theory (DFT) analysis, X-ray diffraction pattern, and X-ray photoelectron spectroscopy (XPS) spectra. In particular, they pointed out that the tandem effect and electronic effect result from the coupling of Ag and Cu nanodomains at their interface (Fig. 2f). Later, Hou et al. further confirmed above conjecture with Ag nanoparticle embedded Cu nanoporous hybrid arrays as model via theoretical calculations [81]. The modified Ag NPs could regulate the electron structure of porous copper at the Cu/Ag interface, which is in favor of the first process of electron transfer to form *CO, and facilitate the adsorption and conversion of *CO to ethylene.

Recently, the researches on atomic scale have received more attention, which partly is ascribed to an essential role of atoms at (or around) the interface, and more importantly, it is conducive to deep insight about electrocatalysis mechanism [76]. Jiao et al. for the first time, proposed atom-pair catalyst for CO_2_RR with FE of CO up to 92% and almost completely suppressed HER [94] and offered a novel and efficient method to construct functional atomic interface at atomic level. The obtained atom-pair catalyst possessed stable Cu_1_^0^–Cu_1_^*x*+^ pair structures, in which Cu_1_^0^ adsorbs CO_2_ and neighboring Cu_1_^*x*+^ adsorbs H_2_O. Based on researches on combining Cu^0^ and Cu^*x*+^ as catalysts for CO_2_RR reported recently [87, 107], Bai et al. [108] systematically studied the reaction path and proposed a possible mechanism to convert CO_2_ to CH_3_CH_2_OH at the interface between Cu^0^ and Cu^*x*+^ with the as-prepared g-C_3_N_4_ supported tetrahedral (Td) Cu_4_ cluster (Td-Cu_4_@g-C_3_N_4_) as catalyst. As shown in Fig. 2g, owing to higher binding energy, CO_2_ molecules tend to be captured by top site of Cu (Cu_t_^0^) at Cu–Cu atomic interface and then reduced to *CO. The clustered *CO induced by the oxidized Cu_b_^*x*+^ (bottom site of Cu) can be further reduced into *CHO. Subsequently, due to lower reaction free energy, the formation of *OHC–CHO* through C–C coupling takes precedence rather than forming two separated *CHO. Finally, ethanol is obtained after multi-proton–electron transfer process. By applying the excellent activity of Cu^+^ to bind *CO, Daiyan et al. constructed Cu^+^/Cu^2+^ interface on the Cu sandwich electrode [109]. The results indicate that the Cu^+^/Cu^2+^ interface is closely related to the conversion of CO_2_ to hydrocarbon, for it can protect Cu_2_O species against being reduced during CO_2_RR. More importantly, the Cu/Cu_2_O interface has also been verified to play an important role in photoelectrocatalysis [90]. A photoelectrochemical cell was constructed with TiO_2_ as photoanode, which could improve the stability of Cu_2_O at the interface via highly energetic electrons and large potential.

In addition, the interface between Cu and other transition metals (such as Sn [85, 110] and Mo [84]) has also been investigated. Owing to the advantages of low cost, low toxicity, and high selectivity to formate [111–114], Sn has been preferably employed to modify copper-based catalysts [115–119]. Recently, the role of Cu/Sn interface has been investigated in detail [85, 109]. For example, Li et al. pointed out that catalytic performance might be affected by the density of Cu/Sn interface. Later, the research conducted by Li et al. suggested that regulating effect of residual Cu^+^ species at the Cu/Sn interface can give rise to an optimal concentration of Sn^2+^ and Sn^4+^ [110], which is prone to formate production [120]. Zang et al. constructed Mo_8_/Cu heterostructures, and the as-obtained catalysts demonstrated FE of 48.68% toward acetate and current density of about 110 mA cm^−2^ at − 1.13 V versus RHE. The intrinsic synergetic effect between Mo_8_ and Cu at Cu–O–Mo interface was substantiated by the experimental and theoretical results [84].

**Fig. 3:**
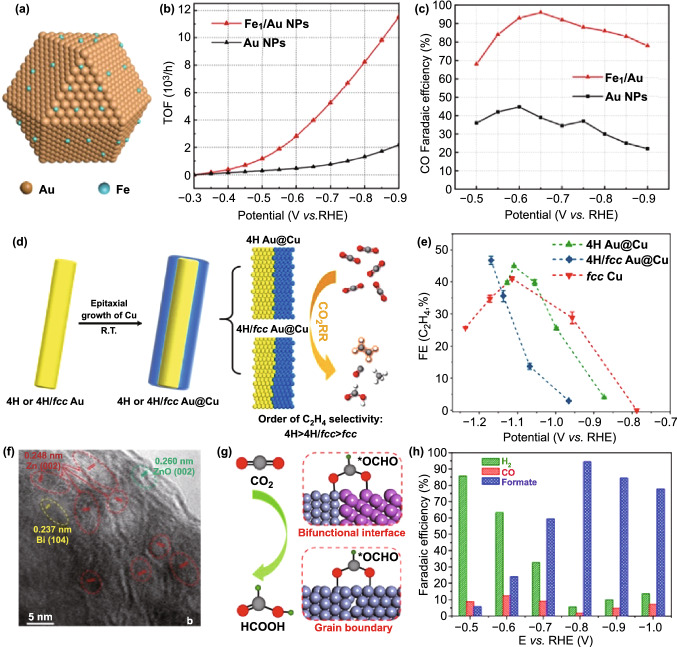
**a** Schematic diagram of atomically dispersing Fe species on Au NPs. **b**, **c** TOF and FEs of CO for Fe_1_/Au and Au NPs. Reprinted with permission from Ref. [122]. **d** Schematic illustration of epitaxial growth of Cu on 4H or 4H/fcc Au nanostructures and their CO_2_RR performance. **e** FEs of C_2_H_4_ on three catalysts. Reprinted with permission from Ref. [127]. **f** High-revolution transmission electron microscope (HRTEM) image of Zn–Bi catalyst. **g** Schematic illustration of catalytic mechanism of Zn–Bi catalyst. **h** FEs of H_2_, CO, and formate for Zn–Bi catalyst. Reprinted with permission from Ref. [130]

Apart from Cu, Au has also been widely studied as excellent catalyst, which is liable to generating CO. On account of the high cost of Au, the cheap and plentiful metals are employed as the second metal to reduce the usage amount of Au by constructing Au–metal interface while maintaining or improving catalytic activity. For example, Back et al. investigated Au/Cu interface for CO_2_RR, demonstrating the importance of metal–metal interface [101]. Later, Kim et al. elucidated the significance of electronic effect on the Au/Ti interface for enhancing CO_2_RR performance [121]. Very recently, Shen et al. built up Fe/Au interface by dispersing isolated Fe atoms on Au NP (Fig. 3a) and tested its electrochemical performance for CO_2_RR. The dynamic changes of catalyst were monitored by operando synchrotron radiation spectroscopies [122]. The atomical dispersion of Fe species on Au NPs was verified by microscopy analysis and X-ray absorption fine structure (XAFS) measurements. As shown in Fig. 3b, c, FE of CO for Fe_1_/Au reached up to 96.3% at 0.65 V versus RHE with mass activity of 399 mA mg^−1^, and the TOF of 11,521 h^−1^ at − 0.9 V versus RHE, significantly exceeding that of pure Au. Operando/in situ characterization technique indicated that the enhanced interaction between Fe and Au atoms at the interface promotes charge transfer from Au to Fe and stabilizes the key intermediates *COOH bonding with the O_2_-phile Fe atom.

Generally speaking, Au tends to be active to produce CO [123–125]. But its selectivity can be adjusted by introducing different second metals or manipulating their crystal phase [99, 104, 126–128]. For example, Chen et al. prepared the high-purity 4H Au@Cu and the heterophase 4H/fcc Au@Cu via the facile epitaxial growth method under ambient conditions (Fig. 3d) [127]. Different from fcc Cu catalyst, the unconventional crystal phase of Cu exhibited higher activity and selectivity to C_2_H_4_. The maximum FE is up to 44.9% at − 1.11 V versus RHE and 46.7% at − 1.17 V versus RHE, respectively (Fig. 3e). Theoretical calculations demonstrate that there is lower energy barrier to form *CHO thus leading to the easier formation of C_2_H_4_ at the 4H phase and 4H/fcc interface of Cu than the fcc Cu. Aimed at illustrating bimetallic interfacial effects, Zhang et al. built the interfacial models of the Cu/Au bimetallic system [129]. DFT calculations suggested the enhancement of CO_2_RR performance is mainly attributed to the bimetallic interface stress and the lower formation energy of H* at the Cu/Au interface than pure Cu. In addition, the Zn/Bi interface has also been studied [130]. The Bi-modified Zn catalyst with metal–metal bifunctional interface (Fig. 3f) and grain boundaries can provide high density of active sites (Fig. 3g), thus achieving high performance with maximum FE of HCOO^−^ up to 94% at − 0.8 V versus RHE (Fig. 3h).

### Metal–Metal Oxide Interface

Among various catalysts for CO_2_RR, the application of most metal oxides has been largely limited due to poor conductivity [131]. An effective strategy of combing metal oxides with highly conductive metal has attracted increasing interest due to not only the improved electrical conductivity, but also the excellent electrochemical performance [132–136]. In fact, this combination is also conducive to improving the performance of metal itself for CO_2_RR [132–134, 137–139]. In brief, the interface between metal and metal oxide synergistically enhances the CO_2_RR performance [133].

**Fig. 4:**
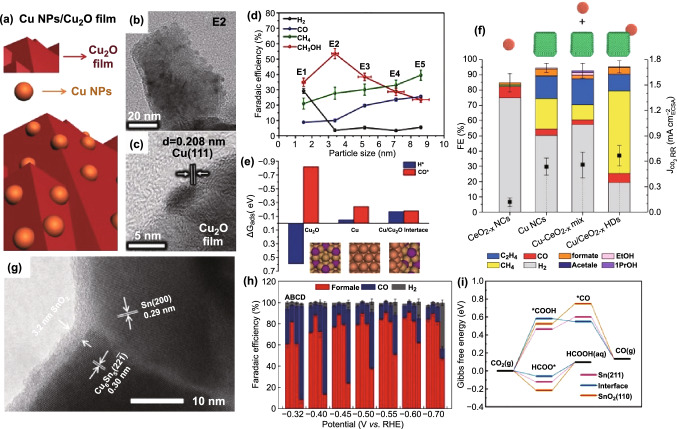
**a** Schematic representation, **b** TEM and **c** HRTEM images of Cu NPs/Cu_2_O film. **d** The dependence of FEs on the Cu NP size. **e** The Gibbs free energies of H* and CO* on different sites. Reprinted with permission from Ref. [90]. **f** FEs and partial current densities for different catalysts. Reprinted with permission from Ref. [141]. **g** HRTEM image of Sn_2.7_Cu catalyst. **h** FEs of formate, CO, and H_2_ for four catalysts. **i** Calculated Gibbs free energy diagrams for CO_2_RR on different catalysts. Reprinted with permission from Ref. [137]

Take the most commonly used Cu-based catalyst [90, 119, 140, 141] as an example, the unidirectional facilitation has been widely studied. For example, Chang et al. built up structurally controlled Cu/Cu_2_O interface (Fig. 4a) via distributing Cu NPs on Cu_2_O film [90].

Importantly, the as-obtained interface offers critical active sites for producing CH_3_OH. Compared with pure Cu NPs and Cu_2_O film, the amount of CH_3_OH rises up significantly at the presence of Cu/Cu_2_O interface. Especially, the maximum FE can reach up to 53.6% at the longest interface (denoting as E2, Fig. 4b, c), which is one of the highest FEs reported using non-noble catalysts [142, 143]. The FE and the amount of CH_3_OH increase with the increasing particle size (Fig. 4d) and interfacial length, respectively. Furthermore, the intrinsic reaction mechanism was investigated with theoretical calculation in terms of thermodynamics and kinetics. Generally, the prerequisite for reducing CO_2_ to methanol at a high ratio requires stronger H* but weaker CO* binding. As shown in Fig. 4e, the introduced Cu NPs helps to strengthen H* and weaken CO* adsorption at the interface and thus facilitates the formation of CH_3_OH. Varandili et al., for the first time, reported the Cu/CeO_*x*_ interface for effective CO_2_RR by surmounting significant lattice mismatch and poor charge conductivity [141]. In particular, the former can be ascribed to the presence of ligands and solid/liquid interface, which can modulate the interfacial energies and thus make it possible to overcome great mismatch and form heterostructures [144]. Compared with simple physical mixture of Cu and CeO_2−*x*_ NCs, the as-synthesized Cu/CeO_*x*_ catalyst with Cu/Ce^*x*+^ interface exhibited 5 times higher FE of 54% toward methane at − 1.2 V versus RHE (Fig. 4f). The theoretical model that a ceria nanoparticle is immobilized on Cu-slab based on Graciani et al. was built to explore the interface effect of Cu/CeO_2−*x*_ [93]. At the microlevel, the enhancement results from bidentate adsorption and stability of CHO* and H_2_CO* on Cu and oxygen vacancies in CeO_2−*x*_, which is conducive to thermodynamically favored pathway toward C_1_ by breaking the CHO*/CO* scaling relationship [145–150]. Afterward, Cu/CeO_2_ catalyst with smaller Cu but more ceria NPs was reported to convert efficiently CO_2_ to ethylene and ethanol [151]. When Cu and CeO_*x*_ NPs were employed to be embedded on carbon nanofibers, the as-obtained Cu/CeO_*x*_@CNFs catalyst exhibited superior activity and selectivity compared to catalysts without Cu/CeO_*x*_ interface [140]. The improvement in catalytic performance can be contributed to the synergistic geometric and electronic effects at the Cu/CeO_*x*_ interface.

Besides, the enhancement of electrocatalytic performance can also be attributed to the interface forming during the test. For instance, Ye et al. reported the in situ reconstructed Sn/SnO_*x*_ interface (Fig. 4g) improved the selectivity toward HCOOH and other C_1_ products [137]. Similarly, the Sn/SnO_*x*_ interface was constructed through the surface reconstruction before CO_2_RR test [120]. As shown in Fig. 4h, among the four catalysts, Sn_2.7_Cu catalyst with Sn-Cu alloy/Sn core and SnO_2_ shell exhibited the highest current density for HCOOH at all measured potentials, and the total current density was up to 406.7 ± 14.4 mA cm^−2^ at − 0.70 V versus RHE. The systematical characterizations of in situ Sn K-edge extended X-ray absorption fine spectra, ex situ XPS spectra and ex situ HRTEM images suggested that the surface SnO_*x*_ lessens significantly, which resulted from partial reduction of the SnO_*x*_ shell on the surface of Sn_2.7_Cu catalyst during the electrochemical test. Meanwhile, under the driving of lower surface free energies, the redundant Sn atoms in core spontaneously can transfer to SnO_*x*_ shell, and thus, the Sn/SnO_*x*_ interface was constructed. Particularly, this phenomenon only exists in Sn_2.7_Cu catalyst owning to the hierarchically heterogeneous Sn-Cu alloy/Sn core structure. Subsequent DFT calculation revealed that the in situ reconstructed Sn/SnO_*x*_ interface can reduce the Gibbs free energy (*G*) via weakening the binding of HCOO* and then facilitate HCOOH production (Fig. 4i). In addition, the high FE of C_1_ production can be partly contributed to superior inhibition of competitive HER, originating from the weakened binding of *H intermediates.

**Fig. 5:**
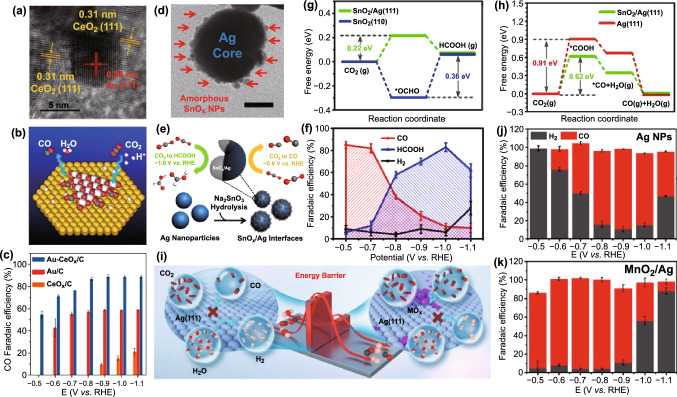
**a** HRTEM image of Au/CeO_*x*_ catalyst. **b** Schematic representation of mechanism of Au–CeO_*x*_/C catalyst. **c** FEs of CO for different catalysts. Reprinted with permission from Ref. [134]. **d** TEM images of SnO_*x*_/Ag NPs. Scale bars: 10 nm. **e** Schematic representation of catalytic mechanism of SnO_*x*_/Ag. **f** FEs for SnO_*x*_/Ag catalyst at different potentials. Free energy diagrams of CO_2_ reduction to **g** HCOOH on SnO_2_ (110) and SnO_2_/Ag (111) and **h** CO on Ag (111) and SnO_2_/Ag (111). Reprinted with permission from Ref. [155]. **i** Schematic representation of CO_2_ to CO on the Ag and MO_*x*_/Ag catalysts. FEs of H_2_ and CO on **j** Ag NPs and **k** MnO_2_/Ag NPs. Reprinted with permission from Ref. [160]

Gao et al. built up Au–CeO_*x*_ interface on carbon substrate via loading Au NPs on CeO_*x*_ NPs (Au–CeO_*x*_/C) (Fig. 5a) [134]. It demonstrated superior electrocatalytic performance to the pure Au and CeO_*x*_ and the mechanism is presented in Fig. 5b. As shown in Fig. 5c, the Au–CeO_*x*_ interface showed higher FE of CO generation at all test potentials compared with the pure ones, and the highest FE is up to 89.1% at − 0.89 V versus RHE. Moreover, the geometric current density of CO on Au–CeO_*x*_/C catalyst is 1.6 times higher than those on the two single-component catalysts. In fact, it has been reported that better performance can be achieved when improving utilization of Au NPs electrostatic adsorption process [152]. Contrast experiments showed that hydroxyl groups from the decomposition of water [153] can not only stabilize Ce^3+^ on the surface to facilitate redistribution of oxygen vacancies from bulk to surface [154], but also improve the stability and absorption of CO_2_^δ−^ species at the interface. At the same time, the Bader analysis suggested the presence of interface and hydroxylation can both lead to the reduction of Ce^4+^. The interaction between Au and Ce^*x*+^ at the interface makes for the increased concentration of Ce^3+^. Theoretical analysis showed that the Ce^3+^ species could stabilize *COOH whose formation is limiting step, via direct interaction with terminal oxygen to improve conversion of CO_2_ to CO. In addition, the catalysts prepared by substituting Au with Ag were also studied, which similarly demonstrated the improvement in catalysis performance. The research, provided significant instruction for the design of metal-CeO_2_ interface as efficient electrocatalysts.

Recently, metal–oxide interface with highly synergistic metal–oxide interactions, for the first time, has been successfully obtained [155]. The as-prepared catalyst consisted of Ag core and SnO_*x*_ decorated on the surface of Ag (Fig. 5d). Different from conventional core–shell structure, the SnO_*x*_/Ag heterostructure simultaneously possesses the exposed Ag and SnO_*x*_ sites, leading to dual function (Fig. 5e). As shown in Fig. 5f, CO and HCOOH are main productions in the more positive and negative potential range, respectively. In addition, it demonstrated much lower overpotential and higher FE of CO, and higher selectivity for HCOOH generation, compared with single-component Ag and SnO_*x*_ NPs, respectively. Importantly, the selective conversion of CO_2_ to different products in different potential ranges can be achieved at the metal–oxide interface. Notably, the SnO_*x*_/Ag heterostructure can avoid CO poisoning and thus present excellent catalytic stability that outperformed Pd, the only known material that can realize switch of HCOOH and CO at different potentials [156, 157]. The theoretical calculations accompanied with comparative experiment were carried out to study the internal mechanism. For the CO formation process, *COOH species are suggested to be more thermodynamically stable on the Ag sites, which can contribute to the additional O-Sn bond from SnO_2_ sites. As for HCOOH, the existence of Ag sites weakens the binding of *OCHO on neighboring SnO_2_ sites, which is conducive to HCOOH desorption. Furthermore, as shown in Fig. 5g, h, lower energy barrier for CO pathway serves as the main driving factor for CO-producing mode; at higher overpotential, more energetically favorable ΔG for the formation of *OCHO leads to HCOOH-producing mode. Besides, it is harder to adsorb H atoms for more positively charged Ag, contributed by the electron transfer from Ag to SnO_*x*_ in the whole potential range.

Inspired by other works [99, 119, 131, 158, 159], Yuan et al. systemically studied Ag-based catalyst with different Ag–metal oxide interfaces, i.e., MO_*x*_/Ag (111) (M = Cu, Cr, Sn, Bi, Pb, Mn) [160]. As shown in Fig. 5i, MO_*x*_/Ag interface can lower Δ*G* for the formation of *COOH and the key *COOH intermediate can be stabilized via additional coordination bonds between carbonyl and M at the interface. Meanwhile, the energy barrier for H_2_ generation is increased and thus HER is significantly suppressed. Figure 5j, k shows that MnO_2_/Ag catalyst exhibits much higher FE of CO at more positive potential range (from − 0.5 to − 0.9 V vs. RHE) than pure Ag NPs. Notably, the FE of CO is up to 98.0% at − 0.7 V versus RHE, and the stability was increased considerably. Apart from these five metals, Mg and Ti oxides have also been investigated via DFT study and the Ag–oxide interface exhibits significant facilitation effect on CO_2_ electroreduction [161, 162]. For the 2D MgO/Ag catalyst, the excellent electrocatalytic performance could be attributed to exotic surface states of MgO overlayers, mediated by electron coupling between MgO and Ag substrates. While for (TiO_2_)_3_/Ag(110) electrocatalyst, the Ti oxides/Ag interface functions by providing active sites for the adsorption and activation of CO_2_ molecule. To be specific, Ag as electron donor could supply electrons to both (TiO_2_)_3_ and the absorbed CO_2_, forming CO_2_* at the interface.

### Metal–Nonmetal Interface

Constructing metal–nonmetal interface is another strategy to improve CO_2_RR efficiencies of metal catalysts [91]. The electron transfer from metal to nonmetal [163–167] triggered by the difference in electronegativity and the atomic level distance at the interface can contribute to the key steps in CO_2_RR. In addition, the metal–nonmetal interface can be further modulated with various nonmetal elements, such as N, to tune the interfacial electron transfer.

**Fig. 6:**
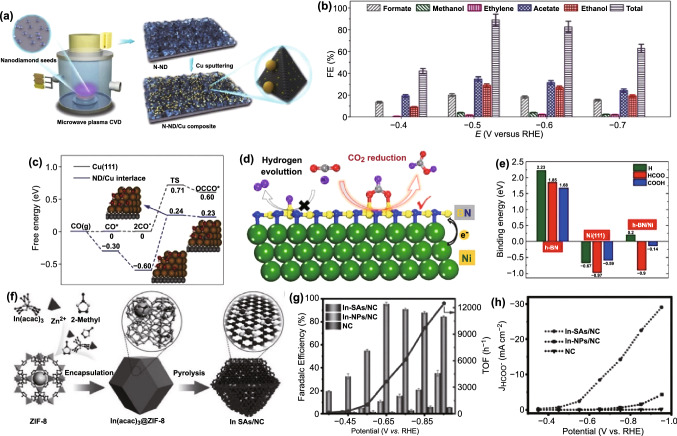
**a** Schematic illustration for N-ND/Cu composite materials. **b** FEs of different products for N-ND/Cu catalyst. **c** Δ*G* diagram for CO coupling at the Cu and ND/Cu interface. Reprinted with permission from Ref. [164]. **d** Schematic diagram for CO_2_RR at h-BN/Ni interface. **e** Binding energies of H, HCOO, and COOH on three catalysts. Reprinted with permission from Ref. [165]. **f** Schematic diagram for synthesis of In-SAs/NC. **g** FEs and TOF of HCOO^−^ and **h** current densities for three catalysts. Reprinted with permission from Ref. [166]

For example, Wang et al. constructed N-doped nanodiamonds/Cu (N-ND/Cu) interface via sputtering Cu NPs on the surface of N-ND (Fig. 6a) [164], which exhibited one of the highest FE of C_2_ products reported [62, 168–176]. As shown in Fig. 6b, compared with sole parent catalytic component, N-ND/Cu catalyzes CO_2_ reduction in a more positive potential range (− 0.4 to − 0.7 V vs. RHE) and possesses higher activity and selectivity for acetate and ethanol. More importantly, the Cu mass activity as well as the unprecedented persistent catalytic performance up to 120 h at − 0.5 V versus RHE was achieved. According to double-layer capacitance measurements [177–179] and other analysis (grazing-incidence wide-angle X-ray scattering, SEM, etc.), the good durability is ascribed to the synergistic stabilization of N-ND/Cu interface. DFT calculations were carried out to reveal the possible intrinsic mechanism to shed light on the high activity and selectivity. Different from other reports [180, 181], they concluded that the charge transfer from Cu to diamond that results from subsurface O can strengthens CO binding at the interface which can further be facilitated by N doping. Therefore, as illustrated in Fig. 6c, the desorption of CO was suppressed and the dimerization of CO to *OCCO was enhanced, which is dynamically more favorable at the interface. In addition, the role of N doping can not only improve the conductivity of ND and the stability of Cu, but also lower energy barrier for formate generation with high selectivity.

Beside electronic effect, geometrical structure is also of vital importance [165, 182]. The h-BN monolayers were coupled with transition metals to develop CO_2_RR electrocatalysts. Furthermore, DFT has been employed to investigate of the origin of activity of h-BN/metal for CO_2_RR [165] through establishing the relationship between the optimized geometries and the different intermediates (such as H, HCOO, and COOH). As illustrated in Fig. 6d, the abundant electrons transfer from the metal to h-BN should predominate the conversion of CO_2_ to HCOOH while HER is suppressed. Moreover, Fig. 6e clearly shows that at the h-BN/Ni interface, the binding energy of H and COOH is greatly weaken, while the change in HCOO binding is slight.

Over the past decade, more attention has been focused on single-atom catalyst due to its appealing electrocatalytic performance as a result of almost 100% atomic utilization [183–189]. However, a recent research showed that metal cluster catalyst with the atomic interface outperforms the corresponding single-atom catalysts [190], implying that the well-defined atomic interface can deliver outstanding catalytic performance. Very recently, the indium (In) single-atom catalyst with N-doped carbon matrix (In-SAs/NC) was prepared via a wet-impregnation process and subsequent a pyrolysis process, which possessed exclusive In^δ+^-N_4_ atomic interface on MOFs derived N-doped carbon matrix (Fig. 6f) [166]. Compared with In nanoparticle catalyst (In-NPs/NC) and NC, Fig. 6g, h shows prominent enhancement on FE and current density of formate when In-SAs/NC was employed as catalyst, which was also well explained by DFT calculations. Notably, the FE and the current density of formate are up to 96% and 8.87 mA cm^−2^ at − 0.65 V versus RHE, respectively. Furthermore, the strategy can also be extended to other two group metals (Sn and Sb), and relevant single-atom catalyst both demonstrated excellent catalytic performance. The obtained Sn-SAs/NC and Sb-SAs/NC catalysts showed the maximum FE of 88% toward HCOO^−^ at 0.75 V versus RHE and above FE of 80% in broad potential windows (− 0.65 to − 0.95 V vs. RHE), respectively. That is, they provided a new strategy with universality to construct main group metal single-atom catalysts with outstanding electrocatalytic performance for CO_2_RR.

### Metal Oxide–Metal Oxide Interface

**Fig. 7:**
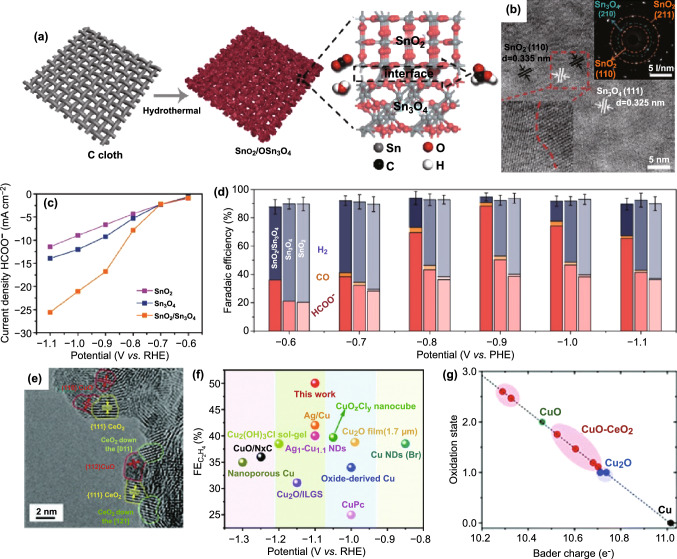
**a** Schematic illustration of the synthetic process for the heterophase SnO_2_/Sn_3_O_4_ nanosheets. **b** HRTEM image of SnO_2_/Sn_3_O_4_. The inset is selected area electron diffraction pattern. **c** Partial current densities of HCOOH and **d** FEs of HCOOH, CO, and H_2_ for SnO_2_/Sn_3_O_4_, SnO_2_ and Sn_3_O_4_. Reprinted with permission from Ref. [194]. **e** HRTEM image of CuO–CeO_2_. **f** FEs of C_2_H_4_ for CuO-CeO_2_ catalyst and other reported Cu-based electrocatalysts. **g** Bader charge analysis about oxidation states of surface Cu atoms. Reprinted with permission from Ref. [197]

According to previous works, the interface of metal oxide and metal oxide can enhance the adsorption and activation of CO_2_ and the stabilization of CO_2_^−^ intermediate on the surface of catalysts [191–194]. In addition, this electronic effect can also contribute to the formation and stabilization of active oxidation state species at the metal oxide–metal oxide interface [120, 195, 196]. Especially, strong electron transport or charge redistribution is highlighted, such as in Sn oxides [193, 194]. For example, electron transfer at SnO_2_/Sn_3_O_4_ interface was investigated by Wu and co-workers [194]. SnO_2_/Sn_3_O_4_ interface was obtained via a facial hydrothermal process and the as-obtained catalyst demonstrated apparent enhancement of CO_2_RR (Fig. 7a, b). Both the current density and FE of formate for SnO_2_/Sn_3_O_4_ catalyst are higher than those of single-component catalysts (Fig. 7c, d). Note that the highest FE of formate is up to 88.3% at − 0.9 V versus RHE. The experimental and theoretical results suggest that the excellent CO_2_RR activity and selectivity should come from a built-in electric field at heterophase interface. More, the built-in electric field can reform electronic structure for CO_2_ adsorption and HCOO* formation and facilitate electron transfer leading to fast reaction kinetics. It is the strong charge redistribution at interface that favors to keep Sn^II^ species (active for HCOOH) abundant and stable. More details about electron transport were studied about SnO_2_/Bi_2_O_3_ interface later [193]. The electron transfer from Bi_2_O_3_ to SnO_2_ endows SnO_2_ with rich electron and prevents itself from reduction for excellent durability. In addition, the introduced Bi_2_O_3_ can help to absorb HCOO* and convert reactant molecules to HCOO^−^.

In addition, studies about the interface of CuO with other metal oxides, especially with CeO_2_, have been reported. For example, Cu-based catalyst with abundant CuO/CeO_2_ interfaces (Fig. 7e) was obtained regardless of mismatched lattices [197], which can catalyze CO_2_ to ethylene with extremely high FE of 50.0% at − 1.1 V versus RHE, outperforming many recently reported Cu-based materials (Fig. 7f). Figure 7g shows that CeO_2_ can change the oxidation state of Cu^2+^(CuO) to Cu^+^ which is crucial for reduction of CO_2_ to C_2+_ products [198, 199]. In this case, CeO_2_ severs as impetus for water activation in CO_2_ reduction, which is kinetically in favor of the formation of *CHO and further formation of C_2+_ [200]. As for insight into how CeO_2_ stabilizes the crucial Cu^+^ species, i.e., specific electron transfer, it still needs to be further explored.

### Organic Molecules–Inorganic Materials Interface

**Fig. 8:**
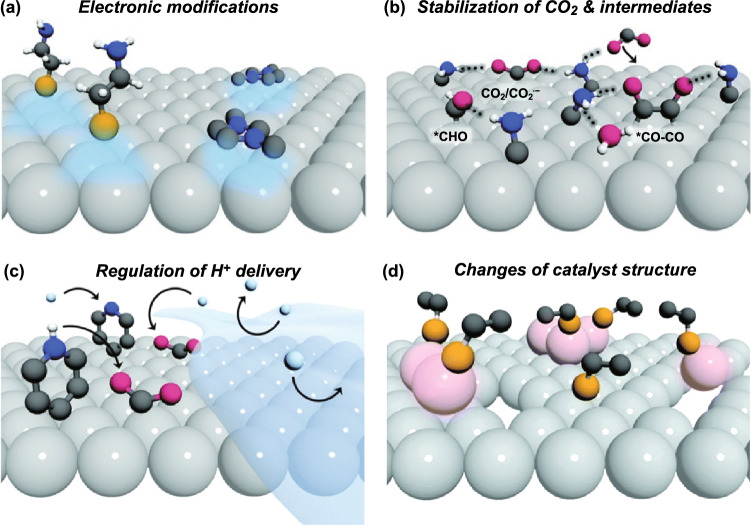
Schematic illustration of the potential roles of organic molecules in modifying the activity and selectivity of inorganic materials for CO_2_RR. Reprinted with permission from Ref. [201]

Herein, the CO_2_RR catalysts of organic molecule-modified inorganic materials are defined as surface coordination chemistry involved nanocomposite structures. This structure investigates how the type and the coordination of ligands influence catalytic activity of the inorganic materials at the molecular level. The surface ligand can not only lead to shape-controlled synthesis, but also trigger many fantastic surface properties of inorganic materials. Appropriate surface coordination chemistry has been reported to provide steric interactions and electronic modifications to the inorganic materials for promoting the catalytic activity. The current difficulty in the investigation of organic molecule-modified inorganic materials lies in the lack of appropriate and effective testing tools to visualize the molecular surface coordination structures. Generally speaking, the molecular mechanisms in surface coordination chemistry can be summarized in two important impacts of surface ligands effect and support effect. Specifically, the role of organic molecules in the study of CO_2_RR can be summarized in the following four categories (Fig. 8): (i) modification of the electronic structure of inorganic materials, (ii) stabilization of the key reaction intermediates, (iii) regulation of mass diffusion (proton/CO_2_), and (iv) modulation of the structural transformation.

#### Organic Molecule-Modified Cu

**Fig. 9:**
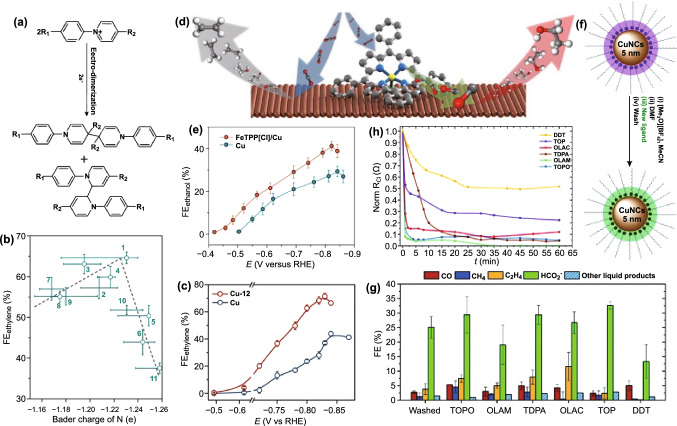
**a** The process of converting N-arylpyridinium salt to mixture of N-aryl-substituted tetrahydro-bipyridines. **b** Relationship of FE of ethylene and calculated Bader charge for the nitrogen atom of the N-aryl-substituted tetrahydro-bipyridines. **c** FEs of ethylene on Cu and Cu-12. (12: *N*,*N*′-(1,4-phenylene) bispyridinium salt). Reprinted with permission from Ref. [202]. **d** Schematic illustration of molecular complexes on the Cu surface. **e** FEs of ethanol for the FeTPP[Cl]/Cu and Cu catalysts. Reprinted with permission from Ref. [203]. **f** Overview of preparing the CuNC catalysts with different ligands. **g** FEs of different reduction products. **h** Changes in the charge-transfer resistance (*R*_CT_) over time for CuNCs capped by different ligands. Reprinted with permission from Ref. [204]

It is well known that Cu is one of the few metals that can reduce CO_2_ to C_2+_ hydrocarbon products. However, the low selectivity of Cu is currently the main challenge for producing economically desirable hydrocarbons with high selectivity. The local environment has been proved to be very important in electrocatalysis through modulating the interactions among reactants or intermediates. Different strategies have been developed to optimize the Cu catalysts through morphology control, grain boundaries design, facets tuning, oxidation state modulation, and dopant manipulation. The above methods have demonstrated favorable and desirable reaction pathway by tuning the stabilities of intermediates for improving selectivity. Unfortunately, the FE of ethylene for Cu catalysts is still unsatisfactory with a low energy efficiency. Recently, the research developed a molecular tuning strategy in which N-arylpyridinium-derived film was used to functionalize the surface of Cu-based electrocatalysts (Fig. 9a) [202]. Moreover, different types of molecules arising from electro-dimerization of arylpyridiniums have been investigated through systematic electrochemical test, operando/in situ spectroscopic study, and computational work. The Bader charge result signified a volcano-shaped trend relationship with FE of C_2_H_4_ (Fig. 9b), and the tetrahydrobipyridine that has suitable electron-donating ability showed a highest selectivity to C_2_H_4_. The result verified that the presence of organic molecules film on the surface of Cu can stabilize an ‘atop-bound’ CO intermediate, thereby leading to a high ethylene generation with a high FE of 72% (Fig. 9c), a high full-cell energy efficiency of 20%, and a good stability of 190 h. In addition, the molecule–metal catalyst interface was also cooperatively designed in order to generate local environment with rich reaction intermediates. Specifically, the Cu surface was functionalized with a library of porphyrin-based metallic complexes (Fig. 9d) [203]. Note that the adsorbed metallic complexes can catalyze the conversion of CO_2_ to CO molecules. Aided by in situ/operando characterizations of Raman and X-ray absorption spectroscopies together with density functional theory calculations, it was verified that the surface metallic complexes can induce a high local concentration of CO intermediates, which can manipulate the C–C coupling through the ethanol pathway. The resultant FE for the conversion of CO_2_ to ethanol is 41% (Fig. 9e), and the overall energy efficiency is 13%. The synthesis of colloidal nanoparticles inevitably requires organic ligands to control the size and morphology of the nanostructures. In this way, the structure–activity relationships of the catalysts can be well identified. However, the dynamic evolution of catalyst surface ligands in electrocatalysis and their role in the catalytic process are still unclear. The research used Cu nanoparticle as benchmark catalysts to investigate the effects of capped different organic ligands (Fig. 9f) (i.e., oleylamine (OLAM), oleic acid (OLAC), dodecanethiol (DDT), trioctylphosphine (TOP), trioctylphosphine oxide (TOPO), and tetradecylphosphonic acid (TDPA)) on the CO_2_RR activity [204]. The selection of ligands is based on the following three reasons, i.e., wide range of applications, varied binding strength derived from different functional groups, and a typical research case for studying the correlations between catalytic activity and ligand surface coverage. At the reaction potential, DDT can behave as a stable ligand on the surface of Cu, while TOPO, OLAM, TDPA, and OLAC are labile and will not affect the final selectivity and the activity of catalysts (Fig. 9g). TOP functions as a watershed to judge whether the ligand is stable (Fig. 9h). The stability of the surface ligands is binding-strength-related, which is dependent on the applied cathodic potential during electrocatalysis rather than their pristine electroreduction potentials. Based on the in situ observation, only the strongly bound ligands can produce a modulation for the electrocatalytic performance, while the weakly bound ligands were removed quickly at a relatively high cathodic potential. Therefore, aiming at the effective interface design between organic molecule and inorganic material, the research provided a criterion to choose persisted ligands.

#### Organic Molecule-Modified Au

**Fig. 10:**
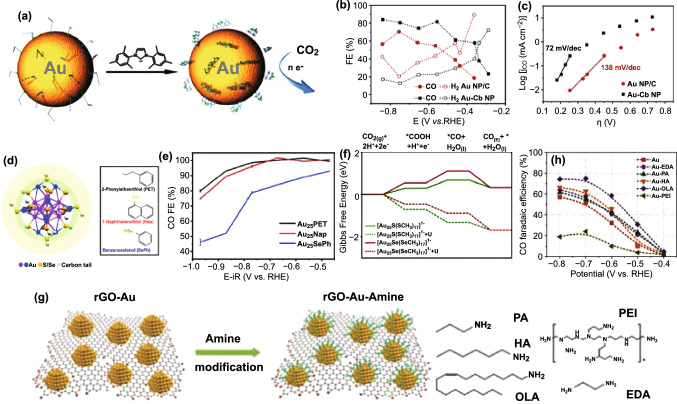
**a** Schematic illustration of preparation and catalysis process of N-heterocyclic (NHC) carbene-functionalized Au NP catalyst. **b** FEs of CO and H_2_ for Au–Cb NP and Au NP/C. **c** Tafel plots of Au–Cb NP and Au NP/C. Reprinted with permission from Ref. [205]. **d** Atom packing structures of Au_25_ and formulas of three ligands. **e** FEs of CO for Au_25_ nanoclusters. **f** Energetics of the CO_2_RR pathway. Reprinted with permission from Ref. [206]. **g** Schematic illustration of amine modification on the rGO-Au composite. **h** FEs of CO for rGO-Au and Au-amine catalysts. Reprinted with permission from Ref. [124]

Furthermore, a molecular of N-heterocyclic (NHC) carbene was used to modify the surface of Au nanoparticle for CO_2_RR (Fig. 10a) [205]. Compared with the pristine Au nanoparticles with a FE of 53% toward CO, the designed catalyst showed a higher FE of 83% at the 0.46 V overpotential (Fig. 10b) with testing condition of neutral pH in water. With current density as an evaluation criterion, the modified Au exhibited about 7.6-fold increase, and meanwhile the NHC carbene-functionalized sample demonstrated a lower Tafel plot (72 mV/decade) than that of bare sample (138 mV/decade) (Fig. 10c). In addition to the strategies of size, shape, composition, and defect control for CO_2_RR catalyst design, the molecular ligand approach can also effectively manipulate the mechanistic pathways toward higher performance.

Ligand effects have also been studied in the case of Au_25_ nanoclusters at the atomic level for CO_2_RR [206]. The protecting ligand are varying from carbon tail to anchoring atom (S or Se) (Fig. 10d). The former (carbon tail) has no visible effect on the resultant catalytic activity and selectivity. In contrast, the latter (anchoring atom) exhibits significant effect on catalytic selectivity. Specifically, the anchoring atom Se tends to accelerate HER, while S can provide a high selectivity to CO (Fig. 10e). The theoretical calculation demonstrated that the energy barriers on S sites for the *COOH/*CO intermediate formations are about 0.26/0.43 eV lower than those of Se, respectively (Fig. 10f). The sulfur sites featured with higher electron density are suggested to be responsible for the bonding difference of the reaction intermediates. Therefore, the anchoring atoms at the metal–ligand interface are required to draw attention in the study of CO_2_RR.

Ultrasmall Au nanoparticles typically have two contradictory properties, namely an abundance of low coordination sites in favor of HER and a high mass activity in favor of CO_2_RR. In order to promote the CO_2_RR efficiency, the ultrasmall Au nanoparticles with a size of 2.4 nm are loaded on reduced graphene oxide (Fig. 10g) [124]. The prepared catalyst exhibited FEs ranging from 32 to 60% (at overpotentials of 450–600 mV) and a high Au-specific mass activity of > 100 A g^−1^ for the conversion of CO_2_ to CO. Interestingly, amine functionalized Au showed an obviously improved efficiencies to 59–75% and the high mass activities are still remained (Fig. 10h). Moreover, the activities of amine-Au catalysts for CO formation are highly dependent on the molecular structure of amine. The branched polyamine is verified to inhibit the CO generation. On the contrary, the linear amines, especially with high alkyl chain length, are beneficial to the formation of CO. The coverage of molecular involving metal–organics interaction and the molecular configuration may contribute to the CO_2_RR.

**Fig. 11:**
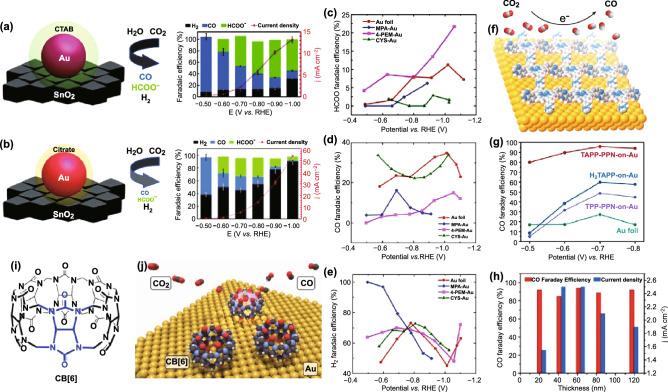
Schematic illustration and catalytic performance of **a** CTAB-Au/SnO_2_ and **b** Cit-Au/SnO_2_ for the electroreduction of CO_2_. Reprinted with permission from Ref. [158]. **c**–**e** FEs of HCOO^−^, CO, and H_2_ for three thiol-tethered ligands functionalized Au nanoparticles. Reprinted with permission from Ref. [207]. **f** Schematic illustration of catalysts with MDI. **g** FEs of CO for TAPP-PPN-60-on-Au, TTP-PPN-on-Au, H_2_TAPP-on-Au, and Au foil. **h** FEs of CO and total current density of TAPP-PPN-on-Au related to the thickness at − 0.7 V versus RHE. Reprinted with permission from Ref. [208]. **i** The structure of CB[6]. **j** Schematic illustration of CO_2_ conversion to CO within the cavity of CB[6] adsorbed on Au surface. Reprinted with permission from Ref. [210]

The organic ligand capped Au showed a significant impact on the metal–oxide interactions (Au/SnO_2_) for CO_2_RR [158]. With cetyltrimethylammonium bromide (CTAB) as capping ligand, the Au endows the interface of Au/SnO_2_ with CO generation at more positive potential and HCOO^−^ formation at more negative potential, respectively (Fig. 11a). In contrast, when Au is capped with citrate, the Au/SnO_2_ catalyst showed a high electivity to H_2_ production among all potential (Fig. 11b). It indicated that the capping ligand can also modulate the metal–oxide interface for CO_2_RR.

Three thiol-tethered ligands (2-mercaptopropionic acid, 4-pyridinylethanemercaptan, and cysteamine) functionalized Au nanoparticles were prepared to demonstrate the relationship between functional ligands and the CO_2_RR selectivity (Fig. 11c–e) [207]. Compared with the pristine Au foil, the FE and the formate production of Au electrode capped with 4-pyridinylethanemercaptan delivered approximately two and threefold enhancement, respectively. Both promotion in CO and H_2_ production was observed in cysteamine-modified Au electrode. The 2-mercaptopropionic-capped Au electrode exhibited almost 100% FE of H_2_. The difference of the three types of ligands in p*K*_a_ is proposed to lead to varied proton-involved desorption mechanism, thus responsible for the dramatic change in the CO_2_RR selectivity.

Moreover, molecularly defined interface (MDI) between Au and tetrakis-5,10,15,20-(4-aminophenyl) porphyrin (H_2_TAPP) was constructed by precisely tuning the voltammetry cycle times in electrochemical oxidation deposition [208, 209]. The abundant Au site and amino functional group at the interface exhibited superior catalytic performance for CO_2_RR (Fig. 11f) with CO selectivity of 95% at a potential of − 0.7 V versus RHE (Fig. 11g). The catalytic performance is verified to be molecular layer thickness-dependent (Fig. 11h). Specifically, with the growing of the thickness of TAPP-PPN, the number of active sites at the MDI increases while the CO_2_ diffusion pathways are blocked. The two inverse trends come to equilibrium when the thickness is 60 nm, which is signified to be beneficial to CO_2_ diffusion kinetics.

A hybrid organic–inorganic catalyst of macrocycle cucurbit[6]uril (CB[6]) (Fig. 11i)-modified Au was developed to the control over the formation and the stabilization of reaction intermediates through engineering surface active sites of Au (Fig. 11j) [210]. The hydrophobic cavity of CB[6] is verified by surface-enhanced infrared absorption (SEIRA) spectroscopic experiment to increase the local CO_2_ concentration close to the surface of Au in the testing condition of KHCO_3_ aqueous solution. The experimental results of the difference in current densities of CO and H_2_ generation indicated that the interaction form of intermediates inside and outside the cavity is different, thereby suggesting an important methodology and mechanistic insight for organic molecule-modified inorganic materials to steer the reaction intermediates through interfacial host–guest chemistry.

#### Organic Molecule-Modified Ag

**Fig. 12:**
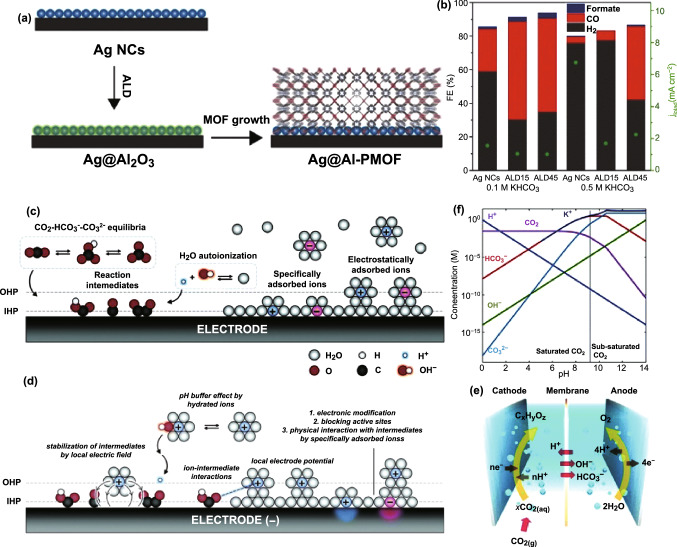
**a** Scheme illustrating the synthesis of Ag@Al-PMOF hybrids. **b** FEs and total current densities for Ag NC and Ag@Al-PMOF catalysts. Reprinted with permission from Ref. [211]. **c** Schematic illustration of the electric double layer composed of IHP and OHP with chemical equilibria involved. **d** Schematic illustration of possible effects of the interfacial ions on the catalyst surface or the electrocatalytic process under the CO_2_RR conditions with the negative potential applied. **e** Schematic illustration of the H-type cell for CO_2_RR. Reprinted with permission from Ref. [201]. **f** Concentration of CO_2_, H_2_, OH^−^, HCO_3_^−^, CO_3_^2−^, and K^+^ as a function of bulk pH of the KHCO_3_/CO_3_^2−^ electrolyte. Reprinted with permission from Ref. [214]

Electronic effects at the interface between Ag nanoparticles and Al-PMOF ([Al_2_(OH)_2_-(TCPP)]) (tetrakis (4-carboxyphenyl) porphyrin (TCPP)) were studied [211]. In this research, the native ligands on the surface of Ag were removed by the wrapping of MOF, thus providing an intimate contact at the Ag and MOF interface (Fig. 12a). The H_2_ generation was drastically inhibited while the CO production was promoted in comparison with the pure Ag nanoparticles (Fig. 12b). Furthermore, the combination of MOF with Ag can obviously increase the stability of metal. The electron transfer from the Al-PMOF to the Ag nanoparticles, i.e., electronic effects, is suggested to be responsible for the selectivity promotion in CO_2_RR, while the porous MOF layer-induced mass transport effects contribute a little to the promotion in the activity of CO_2_RR.

### Electrode and Electrolyte Interface

Although there has been considerable progress in CO_2_RR, the understanding about the interface between the electrode and electrolyte remains poor and requires further efforts to elucidate many details, including ionic distribution, pH changes, the kinetics as well as the reaction barriers [201, 212, 213]. When involving solid–solution interface, it is perhaps easier to visualize an electric double layer at the interface of electrode and electrolyte, which is central to electrochemistry and governing external observations of electrochemical reactions. Typically, the reaction intermediates exist in the inner Helmholtz plane (IHP) by chemical bonding, while the hydrated ions lie in the outer Helmholtz plane (OHP) through electrostatic force (Fig. 12c, d). The type and number of ions and the pH (Fig. 12e, f) of the electrolyte can potentially affect the dynamic equilibria of H_2_O and CO_2_ at the interface [201, 214]. As a result, the different ions adsorbed on the surface of electrode can modulate the resultant CO_2_RR process through multiple potential ways, such as interacting with the reaction intermediates, changing the morphological or electronic structure of the top surface of electrode, shielding some specific sites, and so on. However, at present, compared with the well-understood role of heterogeneous catalysts, the impact of electrolytes during CO_2_RR always has been proposed only relying on the observed experimental performance and the simulation results [123], which has been suffering from insufficient understanding. The adequate characterization techniques are highly desired to drive forward the understanding about the role of electrolyte by monitoring the interactions of mass diffusion and chemical balancing at the interface of electrode and electrolyte.

#### Effects of Cation Size in Bicarbonate Electrolyte

**Fig. 13:**
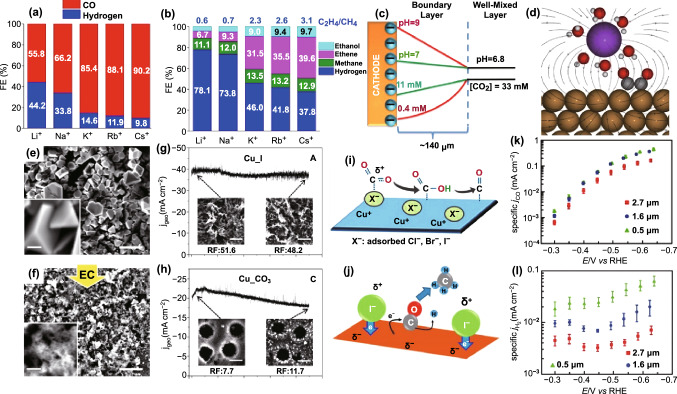
**a** FEs of CO and H_2_ for Ag. **b** FEs of C_2_H_5_OH, C_2_H_4_, CH_4_, and H_2_ for Cu. **c** Distribution of pH and CO_2_ concentration in the boundary layer. Reprinted with permission from Ref. [225]. **d** Schematic illustration of the local electric field created by cation at the catalyst interface and stabilized OCCO intermediate. Reprinted with permission from Ref. [259] SEM images of Cu foils tested before **e** and after **f** the CO_2_ electroreduction. Reprinted with permission from Ref. [233]. Time-dependent geometric current densities of gas products for **g** Cu_I and **h** Cu_CO_3_ catalysts. Reprinted with permission from Ref. [235]. **i** Illustration of facilitation and stabilization of CO_2_ adsorption and carboxyl intermediate, respectively. Reprinted with permission from Ref. [233]. **j** Illustration of how halide affects the net charge of Cu. Reprinted with permission from Ref. [232]. Current densities of **k** CO and **l** H_2_. Reprinted with permission from Ref. [255]

Bicarbonate is widely used as electrolyte for CO_2_RR because it can not only offer near-neutral pH but also increase dissolved CO_2_ concentration [215–219]. The size of cation in bicarbonate electrolyte has been verified to alter the activity and the selectivity in CO_2_RR (Fig. 13a, b) by the relatively high aggregation of cations at the OHP due to cathodic reaction with negative potential [220–222]. The different views about the role of cation size are summarized as below: (i) The large cations are suggested to have relatively small hydration numbers in comparison with small ones, which has a tendency to be readily adsorbed on the electrode [220]. (ii) The cations at the OHP could upgrade the local potential, which are demonstrated to inhibit HER process by decreasing the local proton concentration [223]. The large cations could induce an increased interfacial dipole field in comparison with the small ones, which may stabilize the critical reaction intermediates (e.g., *CO_2_, *CO, and *OCCO) with high dipole torque for producing formate, C_2_H_4_, and C_2_H_5_OH. (iii) The interfacial pH is suggested to be cation-size-dependent by in situ ATR-SEIRAS monitoring the interfacial concentration ratio of CO_2_ and HCO_3_^−^ [218, 224–226]. The p*K*_a_ value of large-size Cs^+^ is evaluated to be about three times lower than that of small-size Li^+^. Compared to Li^+^, Cs^+^ can function as a buffer and increase the local CO_2_ concentration at the electrode–electrolyte interface by a factor of approximately 28 (Fig. 13c). As for the cations with different chemical valence, the cations with high valence but small size are suggested to deliver a significant impact for the interfacial field (Fig. 13d) [223, 227, 259]. Note that the transition metal cation with even trace amount, such as Fe^2+^, Zn^2+^, etc., can trigger the deactivation of the working electrode by electrodeposition [228–230].

#### Effects of Anion Size

Despite the significant influence of cations, the role of the anions still cannot be ignored. The buffering capacity of the anions is thought to be an important factor in the regulation of CO_2_RR pathways [231]. The selectivity to C_2_ products involving C_2_H_4_ and C_2_H_5_OH is increased in non-buffering anions, such as SO_4_^2−^ and ClO_4_^−^. Bicarbonate leads to a mixture of C_1_ and C_2_ products with moderate ratio. As for the phosphate, hydrogen is mainly produced at low overpotentials while CH_4_ is dominant production at high overpotentials. Note that the formation of H_2_ and CH_4_ involves the proton transfers, while the other products, such CO, formate, C_2_H_4,_ and C_2_H_5_OH, are independent of the proton supply. The research suggested that the buffering anion can function as a proton donor to modulate the pH altering, thereby promoting the selectivity to H_2_ and CH_4_. In contrast, non-buffering anion can result in an elevated OH^−^ concentration to inhibit the proton transfer, and as a result, the formation of H_2_ and CH_4_ was suppressed. Specifically, the anion halide in electrolyte is suggested to play multiple roles for CO_2_RR: (i) The halide can trigger surface reconstruction involving the roughness altering and the exposure of active Cu (100) plane for C–C coupling reaction during electrochemical cycling or electroreduction (Fig. 13e, f) [232–236]. The surface electronic states of electrode are also proposed to be modulated by the strong interaction between the halide and the electrode (Fig. 13g, h). (ii) The halide can stabilize the Cu^+^ species in Cu-based catalysts by forming Cu-halide composite that can stabilize methylene intermediate radicals thus favoring the formation of C–C bonds [168, 233]. It means that the binding energies of the reaction intermediates can be altered by the adsorption of halide on the surface of electrode. (iii) The halide can enhance the long-term stability (Fig. 13i, j) [235]. The selectivity of Cu-based electrodes to CH_4_, C_2_H_4_, formate, and C_2_H_5_OH was reported to be increased in the presence of halide [232–234]. In addition, the effects of halide on the activity of Ag and Zn have also been investigated for selective CO production [237–242]. The adsorption of CN^−^ and Cl^−^ on Au surface is demonstrated to give a higher current density than the pristine one for CO production [243]. The theoretical calculation result indicates that the *COOH intermediate can be well stabilized by the adsorbed CN^−^ and Cl^−^ species via van der Walls interaction. In contrast, the effect of electrolytes on nonmetallic catalysts has been less well reported, mainly because of the complexity (presumably electric double layer) near electrode/electrolyte interface. Based on the effects of different cations and anions on the boron-doped diamond for CO_2_RR, the formate selectivity was obviously affected by the alkali metal cations and the halide anions, but the trends are different from that in metal electrode, which needs further investigated [244–246].

#### Modulation of Reactants Supply

Moreover, beyond the essential role of catalyst in CO_2_RR, the mass transport of the reactants including proton, H_2_O, and CO_2_ molecules exhibits significant impact on the resultant catalytic activity. The reason should be ascribed to the fact that at ambient condition, i.e., one atom, neutral pH, and room temperature, the low aqueous CO_2_ solubility of about 33 mM suppresses the mass diffusion of CO_2_ [214, 247], thereby limiting the overall catalytic activity for CO_2_RR. The straightforward strategy for improving CO_2_RR activity and selectivity is increasing the gaseous CO_2_ supply at the interface. A new-developed electrode configuration of three-phase interface, i.e., catalyst (solid)–electrolyte (liquid)–CO_2_ (gas), can provide high-concentrated CO_2_ leading to high CO_2_ reduction rates. Recently, different types of electrode design, such as polyethylene polymer-modified Au [248], 1-octadecanthiol-coated Cu dendrite [249], and graphene-coated wrapped Sn [250], have been successfully developed, and they all delivered high current density and high FE for CO_2_RR. As a result, the strategies that can decrease the local proton concentration at the electrode–electrolyte interface are of significance to suppress the competitive parallel HER, thereby promoting CO_2_RR activity and selectivity [251–254]. The well-tuned morphologies of electrode are suggested to induce local changes of pH at the electrode–electrolyte interface. The inverse opal structure of Au with increased thickness can result in a higher surface basicity to suppress partial current density of HER by about tenfold (Fig. 13k) with slight change in FE of CO (Fig. 13l) [255]. The morphology-dependent local pH change can also be demonstrated in the cases of Au and Zn catalysts [256, 257]. The result indicates that the modifications in the catalyst structures can modulate the supply of reactants (i.e., CO_2_, H^+^, and H_2_O) at the electrode–electrolyte interface [258].

### Molecular Catalysts–Electrode Interface

**Fig. 14:**
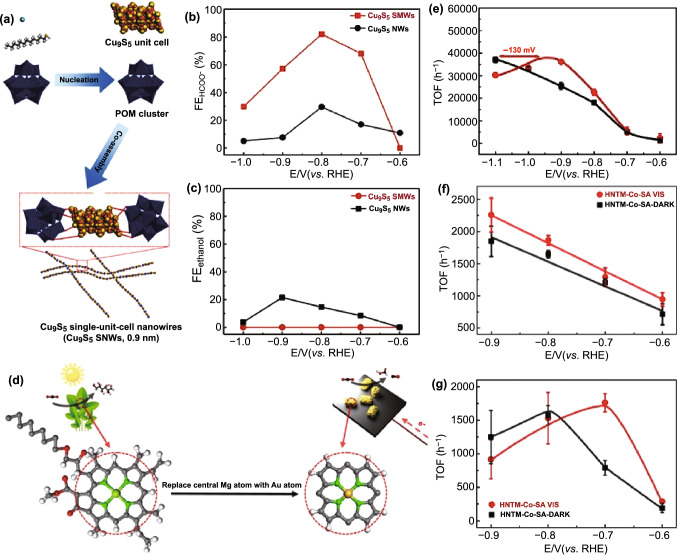
**a** Schematic illustration of preparation of Cu_9_S_5_ SNWs. **b** FEs of HCOO^−^ and **c** FEs of ethanol for Cu_9_S_5_ SNWs and Cu_9_S_5_ NWs. Reprinted with permission from Ref. [262]. **d** Schematic illustration of photosynthesis and photoelectrochemical reduction of CO_2_ on chlorophyll and HNTM-Au-SA. TOF curves of **e** HNTM-Au-S, **f** HNTM-Co-SA and **g** HNTM-Cu-SA under visible light/dark. Reprinted with permission from Ref. [263]

Some important molecular catalysts have been summarized in our recent reviews [73, 260, 261], herein we mainly report the latest findings. Compared with the extensively studied nanocrystal and single-atom catalysts, single-unit-cell catalysts existing as a bridge between the above two catalysts have rarely been reported because of the difficulties lying in synthesis and quantum effects. Recently, by introducing polyoxometalate (POM) cluster, Cu_9_S_5_ single-unit-cell with sub-nanometer of 0.9 nm was prepared through modulating the nucleation pathway (Fig. 14a) [262]. Thus, each unit Cu_9_S_5_ cell can be regarded as molecular catalyst functioning as an isolated active site for CO_2_RR. Compared with the complex productions (HCOO^−^, methanol, and ethanol) of nanocrystal structure, the unit Cu_9_S_5_ cell shows dramatically increased electrocatalytic activity and FE of HCOOH (82.0%) (Fig. 14b, c). The research inspired that the decreasing the size of inorganic nanostructure down to sub-nanometer can achieve great chances for precise catalysis.

Normally, porphyrin serves as a ligand for metal center. A series of porphyrin-Au/Co/Cu catalysts consisting of zirconium porphyrinic metal–organic framework (MOF) hollow nanotubes (HNTMs) as supports and anchored metal atoms were prepared via a solvothermal method and subsequent heat treatment. The porphyrin can mimic the role of chlorophyll as a photoswitch to modulate the electron transfer routes to the metal center (Fig. 14d) [263, 264]. As a result, the light external field can reduce the potential by 130, 20, and 100 mV, respectively, for porphyrin-Au/Co/Cu catalysts (Fig. 14e–g). In addition, organic molecule (such as amino acid)-modified Cu electrodes showed significant enhancement on selectively converting CO_2_ to hydrocarbons [265].

## Conclusion and Perspective

The interface engineering has been developed to be an effective strategy to construct high-performance catalysts toward CO_2_RR. In this review, we have summarized the fundamental and experimental progress in interface engineering for CO_2_RR, which involves metal–metal, metal–metal oxide, metal–nonmetal, metal oxide–metal oxide, organic molecules–inorganic materials, electrode–electrolyte, and molecular catalysts–electrode interfaces, etc. Importantly, the electrocatalytic CO_2_RR performance could be effectively modulated by interface engineering via electronic and/or structural modulation, regulations of electron/proton/mass/intermediates and the control of local reactant concentration, thereby achieving desirable reaction pathway, inhibiting competing hydrogen generation, breaking binding-energy scaling relations of intermediates, and promoting CO_2_ mass transfer. Although great efforts have been devoted to the rational design and controlled fabrication of advanced catalysts with well-defined interfaces, the comprehensive and in-depth understanding of the interface–performance relationship in CO_2_RR still remains a great challenge.

The construction of well-defined interfacial structures is very important to reveal their roles in the structure–performance relationship by the precise control of the interface at the atomic scale. The identification of refined interfacial structures usually requires a delicate combination of aberration-corrected TEM, XAFS analysis and appropriate theoretical simulation methods to confirm the coordination and bonding environment. Moreover, the CO_2_RR involves the adsorption of CO_2_ molecules, multi-electron and proton coupling process, C–C coupling process to generate multi-carbon products, interfacial active species migration or transfer process, and product desorption. The use of operando characterization techniques including but not limited to operando optical, X-ray, and electron-based techniques is highly desired to address some key issues about understanding the role of heterogeneous interface in the stability, the selectivity, and the reaction pathway under real operating conditions. The electrocatalytic tests serve as the dominant evidence to confirm the activity of interface structure. The establishment of accurate and reliable test methods and standards is very important to the development of the field of CO_2_RR. Some pivotal experimental parameters and results, such as the configuration of electrochemical cell, the types of electrodes and electrolyte, the types of gas chromatography columns especially for detection of C_2+_ gas production and so on, should be well described. Theoretical calculations have advanced our understanding of the effect of interface on the CO_2_RR process, and several new concepts and reaction pathways have been proposed. Theoretical calculations of the reaction process are suggested to combine the accurate interfacial structure and the detailed operando characterization, which is of great importance for the design of advanced CO_2_RR catalysts, the revelation of reaction mechanism, etc. In addition, to obtain appreciable reaction rates and conversion efficiencies for CO_2_RR, the electrocatalysis process are suggested to couple with the photo- and/or thermos energy. Thus, the interface should be designed to be active for the synergistic effect of different outer fields. In the near future, the breakthroughs in analysis techniques, data science and artificial intelligence are expected to bring revolutionary progress for the interface-related catalysis in CO_2_RR.
